# Myocarditis and Pericarditis following COVID-19 Vaccination: Inequalities in Age and Vaccine Types

**DOI:** 10.3390/jpm11111106

**Published:** 2021-10-28

**Authors:** Minghui Li, Jing Yuan, Gang Lv, Jacob Brown, Xiangxiang Jiang, Zhiqiang Kevin Lu

**Affiliations:** 1 Department of Clinical Pharmacy and Translational Science, University of Tennessee Health Science Center, Memphis, TN 38103, USA; mli54@uthsc.edu; 2 Department of Clinical Pharmacy and Pharmacy Administration, School of Pharmacy, Fudan University, Shanghai 201203, China; jyuan@fudan.edu.cn; 3 Department of General Surgery, The First Medical Center of Chinese PLA General Hospital, Beijing 100091, China; triangle3000@126.com; 4 College of Pharmacy, University of South Carolina, Columbia, SC 29208, USA; jrb19@email.sc.edu; 5 School of Public Health, Nanjing Medical University, Nanjing 210029, China; xxjiang1125@126.com

**Keywords:** COVID-19 vaccine, mRNA vaccine, myocarditis, pericarditis, age

## Abstract

An increasing number of myocarditis/pericarditis incidences has been reported after coronavirus disease-19 (COVID-19) vaccination in adolescents and young adults. This study was designed to investigate the incidence rate of—and risk for—myocarditis and pericarditis following COVID-19 vaccination in the United States according to age and vaccine type. This study used the Vaccine Adverse Events Reporting System (VAERS) from 11 December 2020 to 13 August 2021. A population-based data mining approach was performed based on the reporting odds ratio (ROR). Adverse events of myocarditis and pericarditis following COVID-19 vaccination were rare, with an incidence rate of 5.98 (95% CI = 5.73–6.24) cases per million doses administered. The incidence rate was higher in adolescents and after the administration of the second dose of messenger RNA (mRNA) vaccines. Overall, two mRNA vaccines were significantly associated with increased risks for myocarditis/pericarditis (mRNA-1273 (Moderna): ROR = 2.91, 95% CI = 2.21–3.83; BNT162b2 (Pfizer–BioNTech): ROR = 5.37, 95% CI = 4.10–7.04) compared to all other vaccines from VAERS. The viral vector vaccine of Ad26.COV2.S (Janssen) was not associated with signals of myocarditis/pericarditis (ROR = 1.39; 95% CI = 0.99–1.97). This study found increased risks for myocarditis/pericarditis following mRNA COVID-19 vaccines. For patients at high risk for myocarditis/pericarditis or with myocardial injuries, the viral vector vaccine may be an alternative for consideration.

## 1. Introduction

Two types of coronavirus disease-19 (COVID-19) vaccines, messenger RNA (mRNA) and viral vector vaccines, are available for adults in the United States. Adolescents aged 12 to 17 years can receive the BNT162b2-mRNA vaccine (Pfizer–BioNTech, New York, NY, USA). All vaccines that are available in the United States have received Emergency Use Authorization (EUA) from the Food and Drug Administration (FDA) [1]. A vaccine that receives EUA is granted authorization from the FDA following a fast-tracked method that ensures the use of a vaccine is safe and effective in humans [2]. Usually, EUA is used in a time of crisis or when a product shows beneficial efficacy, allowing the vaccine to be quickly distributed for use before the full approval. It is expected that the manufacturer of the vaccine granted EUA will continue to provide clinical trial data that support the continued safety of the vaccine. However, the FDA released updated guidance on obtaining EUA for COVID-19 vaccines, stating that the new COVID-19 vaccines must demonstrate a higher standard of safety and efficacy than usual vaccines to be granted EUA [3]. Once EUA is granted, the COVID-19 vaccine must continue to exhibit benefit and safety in both clinical trials and real-world settings to later be fully approved.

The hesitancy to receive COVID-19 vaccination remains high in the U.S. Specifically, about half of young adults are hesitant to be vaccinated [4]. Safety is a driving factor in the public’s trust in the vaccine [5]. As vaccinations are ongoing in the U.S., adverse events have been reported to the FDA and Centers for Disease Control and Prevention (CDC). Many of the reported adverse events are non-serious, such as fever/chills, localized swelling, and localized pain [6]. The prevalence of adverse events, even though most of them are non-serious, causes a significant proportion of the public to have distrust in COVID-19 vaccines [5]. As a result, some adverse events cause the public to be resistant to receiving COVID-19 vaccination. The hesitancy can lead to decreased rates of vaccination, which, in turn, can cause the COVID-19 pandemic to last longer [6].

Adverse events, especially serious ones, are the most common barriers to accepting vaccination [7]. An increasing number of serious but rare myocarditis/pericarditis incidences have been reported after mRNA COVID-19 vaccination in adolescents and young adults [8,9]. Myocarditis is the inflammation of the myocardium (the spectrum); and pericarditis is the inflammation of the pericardium (the inner visceral layer that encloses the heart) [10,11]. Myocarditis and pericarditis can cause heart damage and lead to other serious problems such as gastrointestinal infection or heart failure if untreated [12]. Vaccines causing myocarditis and pericarditis are not new. Rare occurrences of myocarditis and pericarditis were found to be associated with smallpox vaccination [13]. Immunologic reactions after vaccination can cause myocarditis with a predominance of mononuclear cells, direct viral involvement, or cross-contamination [13]. Because myocarditis and pericarditis are associated with the smallpox vaccine, they might be linked to the COVID-19 vaccine through a similar pathology.

The occurrence of myocarditis and pericarditis in adolescents and young adults happens most often after the second dose in the two-series mRNA COVID-19 vaccines [14]. Most myocarditis and pericarditis cases occur within the five days following the second shot of the mRNA COVID-19 vaccine [15]. Adverse events of myocarditis and pericarditis present more often in males of adolescents and young adults [14]. However, most published studies on myocarditis and pericarditis following COVID-19 vaccination are case reports or case series and focus on a specialized population. No population-based studies are available to investigate if COVID-19 vaccines are associated with the increased risks for heart inflammation. While case studies and case series are important in understanding the mechanisms of the link, using population-based big data could allow a better understanding of the association across different groups.

This study aimed to address the gap in the literature and provide a full picture of the risks of COVID-19 vaccines across different groups and, hopefully, provide a strong level of evidence in the safety of vaccination. To better assess the potential risks of COVID-19 vaccines, we conducted a population-based study to investigate the incidence rate of—and risk for—myocarditis/pericarditis following COVID-19 vaccination in the U.S. according to age and vaccine type.

## 2. Materials and Methods

### 2.1. Data Sources

The main data source used in this study was the Vaccine Adverse Event Reporting System (VAERS), a national early warning system to monitor possible safety problems of vaccines [16]. VAERS collects adverse events (possible side effects) after receiving the vaccination. Healthcare providers, manufacturers, patients, and family members can report vaccine-related adverse events to the FDA and CDC. Data collected in VAERS include individual characteristics, vaccines, health care utilization, medications, illnesses, allergies, and adverse events. This study used VAERS from 11 December 2020 to 13 August 2021 to study adverse events after COVID-19 vaccination. The FDA authorized EUA for the first COVID-19 vaccine, the BNT162b2-mRNA vaccine (Pfizer–BioNTech), on 11 December 2020.

The CDC COVID Data Tracker is another data source used in this study [17]. It provides comprehensive information on COVID-19 vaccinations in the U.S., including the dose delivered, the dose administered, the number of people who received at least one dose, and the number of people who are fully vaccinated by region, age, and vaccine type.

### 2.2. Measurement

The vaccines investigated in this study were COVID-19 vaccines. mRNA and viral vector vaccines are two types of COVID-19 vaccines in the U.S. mRNA vaccines include mRNA-1273 (Moderna, Cambridge, MA, USA) and BNT162b2 (Pfizer–BioNTech), and the viral vector vaccine is Ad26.COV2.S (Janssen, Beerse, Belgium). We further studied mRNA vaccines by the first dose and second dose.

Adverse events were coded using preferred terms (PTs) in VAERS. We searched PTs of myocarditis and pericarditis to identify adverse events of myocarditis/pericarditis. The characteristics of the reports examined include age, sex, region, vaccine manufacturer, vaccine dose, the type of adverse event, the number of days to the adverse event, and symptoms after COVID-19 vaccination.

### 2.3. Data Analyses

The incidence rate of myocarditis/pericarditis was estimated using reports of myocarditis/pericarditis divided by the total doses of COVID-19 vaccines administered during the same study period. The incidence rate of myocarditis/pericarditis was further analyzed and reported by age and vaccine type. Individual characteristics, vaccines, adverse events, and symptoms were examined and compared by reports with and without incident myocarditis/pericarditis using Chi-square tests. *p*-values of less than 0.05 were considered statistically significant.

A population-based disproportionality analysis approach was performed based on the reporting odds ratio (ROR) [18]. It is a common approach used in pharmacovigilance studies to identify safety signals. When the ROR is significantly higher than 1.0, the reported adverse events of the drug of interest are significantly higher than the expected adverse events based on all other drugs. We examined whether COVID-19 vaccines were associated with higher likelihoods of reporting myocarditis/pericarditis compared to all other vaccines from VAERS.

## 3. Results

**Figure 1:**
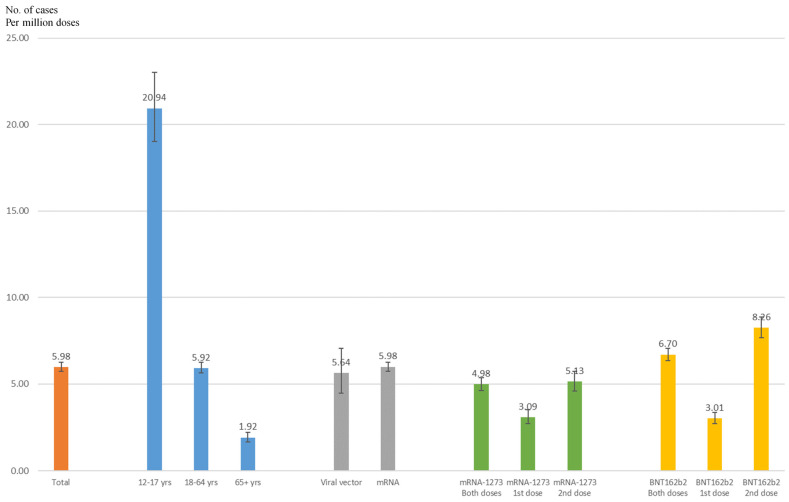
The incidence rate of cases of myocarditis/pericarditis by age and vaccine type.

Adverse events of myocarditis/pericarditis following COVID-19 vaccination were rare, with an incidence rate of 5.98 (95% CI = 5.73–6.24) cases per million doses administered (Figure 1). For different age groups, the incidence rate was the highest in adolescents aged 12 to 17 years, at 20.94 (95% CI = 19.01–23.01) per million, and it decreased with an increase in age to 5.92 (95% CI = 5.62–6.24) per million in adults aged 18 to 64 years and 1.92 (95% CI = 1.65–2.23) per million in older adults aged 65 years and over. For different vaccine types, the incidence rate was higher in mRNA vaccines, at 5.98 (95% CI = 5.73–6.25) per million, than viral vector vaccines, at 5.64 (95% CI = 4.46–7.04) per million. Among mRNA vaccines, BNT162b2 (Pfizer–BioNTech) had a higher incidence rate—of 6.70 (95% CI = 6.34–7.06) cases per million—than the rate of 4.98 (95% CI = 4.62–5.36) cases per million found for mRNA-1273 (Moderna). Notably, the incidence rate following the second dose was twice that of the first dose of mRNA vaccination.

For individual characteristics, cases of myocarditis/pericarditis were more likely to be reported in adolescents (12–17 years) or young adults (18–24 years), males, and those who lived in Northeast or West regions (all *p* < 0.0001) (Table 1). Cases of myocarditis/pericarditis were more likely to be associated with the BNT162b2 (Pfizer–BioNTech) vaccine (*p* < 0.0001) and were more common after the administration of the second dose (*p* < 0.0001).

Myocarditis/pericarditis was more likely to be serious and have emergency room (ER) or office visits than other reported adverse events (all *p* < 0.0001) (Table 1). About 70% of adverse events of myocarditis/pericarditis were serious, 50% had ER visits, and one-third had office visits. Compared to other adverse events, the onset interval between the vaccination date and onset date for myocarditis/pericarditis was longer (*p* < 0.0001). About 50% of cases reported myocarditis/pericarditis two to seven days after vaccination. Chest pain was associated with myocarditis/pericarditis (*p* < 0.0001) and was the most common symptom, accounting for 58.67% of cases of myocarditis/pericarditis. Other symptoms that were significantly related to myocarditis/pericarditis included dyspnoea (*p* < 0.0001), chest discomfort (*p* < 0.0001), palpitations (*p* < 0.0001), and malaise (*p* = 0.0259).

Two types of mRNA vaccines were significantly associated with increased risks for myocarditis/pericarditis (mRNA-1273 (Moderna): ROR = 2.91, 95% CI = 2.21–3.83; BNT162b2 (Pfizer–BioNTech): ROR = 5.37, 95% CI = 4.10–7.04) compared to all other vaccines from VAERS (Table 2). As the only vaccine available for adolescents, the BNT162b2 (Pfizer–BioNTech) vaccine was associated with significant risks for myocarditis/pericarditis (ROR = 8.19; 95% CI = 4.37–15.36) in adolescents aged 12 to 17 years. Both mRNA vaccines were associated with risks for myocarditis/pericarditis in most adult age groups. The risks for myocarditis/pericarditis were double in males compared with females for both mRNA vaccines.

The viral vector vaccine of Ad26.COV2.S (Janssen) was not associated with signals of myocarditis/pericarditis (ROR = 1.39; 95% CI = 0.99–1.97) compared to all other vaccines from VAERS (Table 2). No signals of myocarditis/pericarditis were detected in the Ad26.COV2.S (Janssen) vaccine across different age and sex groups.

## 4. Discussion

This study provides a population-based incidence rate of myocarditis/pericarditis following COVID-19 vaccination. This study used the VAERS to examine reports of myocarditis/pericarditis and the CDC COVID Data Tracker to measure total doses of COVID-19 vaccines administered during the same period. Therefore, the common limitation of passive surveillance data of unknown denominators was solved.

This study shows that myocarditis and pericarditis are rare adverse events of COVID-19 vaccines. Phase III clinical trials of COVID-19 vaccines were not powered to detect such rare events; therefore, myocarditis and pericarditis were not reported in clinical trials of mRNA vaccines. The U.S. population-based incidence rates of myocarditis and pericarditis are 5.73 to 26 cases per 100,000 person-year and 0.95 to 2.16 cases per 100,000 person-year, respectively [19]. Another VAERS study found an incidence rate of 55 cases per 100,000 vaccinees for myocarditis/pericarditis following smallpox vaccination [13]. The incidence rate of myocarditis/pericarditis following the second dose of mRNA vaccines was shown to increase to double that of the first dose. However, both rates are lower than the incidence rate following smallpox vaccination.

Increased risks for myocarditis/pericarditis are only identified in two mRNA vaccines but not in the viral vector vaccine. Specifically, risks for myocarditis/pericarditis are detected in mRNA vaccines in both sexes and most age groups. It is unclear if there is a definitive mechanism in which the mRNA vaccines cause myocarditis/pericarditis. One pathological reason is the autoantibody generation that occurs after receiving mRNA vaccines. Autoantibodies recognize the person’s own cells as foreign pathogens and will attempt to destroy them. One of these cell targets includes cardiac myocytes, which can contribute to the development of myocarditis/pericarditis after receiving mRNA vaccines [15]. Our results, combined with the possible pathology, establish an association between mRNA COVID-19 vaccines and myocarditis/pericarditis.

Among the two mRNA vaccines, BNT162b2 (Pfizer–BioNTech) is associated with a higher overall risk for myocarditis/pericarditis than mRNA-1273 (Moderna). This increased overall risk for myocarditis/pericarditis can be attributed to the use of this vaccine in adolescents. BNT162b2 (Pfizer–BioNTech) is the only COVID-19 vaccine that has received EUA for use in adolescents and is associated with a high risk for myocarditis/pericarditis in adolescents. For other age groups, the risks for myocarditis/pericarditis are similar in mRNA-1273 (Moderna) and BNT162b2 (Pfizer–BioNTech).

Health care providers can use evidence generated from this study to provide consultation on the safety of COVID-19 vaccines. Health care providers should discuss the risks for myocarditis/pericarditis, particularly with individuals at high risk for cardiovascular diseases, to help them to prepare adequately. The benefits and risks of COVID-19 vaccines should be carefully discussed during consultation. It was demonstrated that, in clinical trials, the efficacies of mRNA vaccines were over 90% [20,21]. Adverse events of myocarditis/pericarditis following COVID-19 vaccination were very rare. Most of the patients were fully recovered after vaccine-induced myocarditis/pericarditis. They were able to return to normal daily activities after their symptoms improved. However, certain serious myocarditis/pericarditis might be associated with long-term morbidity [22]. In addition, a modified vaccine schedule or guideline might be needed to address these new adverse events, especially in adolescents and young adults. Individuals with different characteristics were confronted with different risks for myocarditis/pericarditis after COVID-19 vaccination. It is important for health care providers to carefully examine risk factors of myocarditis/pericarditis and weigh benefits and risks when choosing an appropriate type of COVID-19 vaccine. The viral vector vaccine might be an alternative for consideration for individuals at high risk for myocarditis/pericarditis, those who experienced myocarditis/pericarditis events following the first dose of COVID-19 vaccine, or individuals with a family history of cardiomyopathy. Finally, a comprehensive diagnosis and treatment plan might be needed for acute myocarditis associated with receipt of mRNA COVID-19 vaccines.

The main limitation of this study is that the definition of myocarditis/pericarditis is not verified in VAERS. In clinical practice, certain tests (e.g., electrocardiogram, echocardiogram, X-ray, magnetic resonance imaging, or endomyocardial biopsy) are ordered to confirm the diagnosis of myocarditis/pericarditis. In VAERS, however, the reported myocarditis/pericarditis might not be verified by these diagnostic tests. Therefore, the incidence rate and risk for myocarditis and pericarditis identified in this study should be interpreted in consideration of this major limitation.

This study has some other limitations. First, VAERS is a passive reporting system and cases are voluntarily reported. Cases in VAERS can be reported by healthcare providers, manufacturers, patients, and family members. It might contain information that is incomplete and inaccurate. However, vaccine safety experts review all reports of serious adverse events and investigate further for verifications and confirmation when needed. Second, adverse events are usually underreported in VAERS. VAERS receives reports for only a small fraction of actual adverse events; thus, it might not capture all adverse events, which may lead to an underestimation of the associated risks. However, the serious adverse events, such as myocarditis/pericarditis, examined in this study are more likely to be reported than nonserious ones. Last, the causality between the identified vaccine and the described adverse event cannot be established only using VAERS. All reports are accepted by VAERS without judging whether the event is caused by the vaccine, even if the event may be coincidental and related to other causes. This might lead to an overestimation of the associated risks.

## 5. Conclusions

Adverse events of myocarditis/pericarditis following COVID-19 vaccines are rare. This study found increased risks for myocarditis/pericarditis following mRNA COVID-19 vaccines. However, given the current continuity of the COVID-19 pandemic and new variants, we believe that mRNA COVID-19 vaccination should continue to be recommended for those aged 12 years or older. For individuals with myocardial injuries, the viral vector vaccine may be an alternative for consideration. It is essential to continue the benefit–risk assessment and keep the public abreast of any updated findings to maintain public trust in the safety of COVID-19 vaccines.

## Figures and Tables

**Table 1:** Characteristics of reports to the VAERS following COVID-19 vaccination by myocarditis/pericarditis.

	All Reports to the VAERS	Myocarditis/Pericarditis Reports		
		No	Yes	
	%	%	%	*p*
**Individual**				
Age				<0.0001
12–17	3.94	3.86	21.22	
18–24	6.26	6.18	23.77	
25–44	32.95	32.98	27.80	
45–64	34.67	34.74	18.42	
65+	22.18	22.24	8.79	
Sex				<0.0001
Female	71.38	71.58	26.88	
Male	28.62	28.42	73.12	
Region				<0.0001
Northeast	19.49	19.47	23.82	
Midwest	24.13	24.15	18.69	
South	31.71	31.73	27.75	
West	24.67	24.65	29.74	
**Vaccine**				
Type				<0.0001
Viral vector: Ad26.COV2.S (Janssen)	10.21	10.24	3.69	
mRNA: mRNA-1273 (Moderna)	44.20	44.25	33.22	
mRNA: BNT162b2 (Pfizer–BioNTech)	45.59	45.51	63.09	
Dose				<0.0001
1st	65.77	65.90	36.53	
2nd	34.23	34.10	63.47	
**Adverse events**				
Serious	8.17	7.89	69.42	<0.0001
Death	1.29	1.30	0.94	0.1502
Life-threatening illness	1.67	1.60	16.12	<0.0001
Hospitalization	5.90	5.62	66.82	<0.0001
Disability	1.48	1.47	3.53	<0.0001
ER visit	13.62	13.46	48.73	<0.0001
Office visit	19.92	19.85	33.98	<0.0001
Onset interval				<0.0001
0	44.70	44.87	8.52	
1	21.34	21.36	15.98	
2–7	17.47	17.34	45.44	
8+	16.49	16.43	30.06	
**Symptoms**				
Chest pain	2.78	2.52	58.67	<0.0001
Dyspnoea	5.45	5.38	19.93	<0.0001
Pyrexia	15.74	15.74	16.45	0.3714
Chest discomfort	2.20	2.16	11.12	<0.0001
Pain	14.05	14.07	11.03	<0.0001
Fatigue	15.56	15.59	9.90	<0.0001
Chills	14.36	14.39	8.58	<0.0001
Headache	19.26	19.30	8.58	<0.0001
Nausea	11.24	11.27	5.84	<0.0001
Pain in extremity	9.51	9.53	5.18	<0.0001
Myalgia	5.98	5.99	4.81	0.0218
Palpitations	1.99	1.98	4.76	<0.0001
Dizziness	11.55	11.58	4.71	<0.0001
Vomiting	4.28	4.28	4.38	0.8214
Malaise	2.91	2.91	3.72	0.0259
Arthralgia	6.06	6.08	3.53	<0.0001

VAERS: vaccine adverse event reporting system; COVID-19: coronavirus disease-19; mRNA: messenger ribonucleic acid; ER: emergency room.

**Table 2:** Myocarditis/pericarditis following COVID-19 vaccination compared to all other vaccines from VAERS by age and sex.

	Viral Vector			mRNA					
	Ad26.COV2.S (Janssen)			mRNA-1273 (Moderna)			BNT162b2 (Pfizer–BioNTech)		
	Cases	ROR	95% CI	Cases	ROR	95% CI	Cases	ROR	95% CI
**Overall**	78	1.39	(0.99–1.97)	703	2.91	(2.21–3.83)	1335	5.37	(4.10–7.04)
Age									
12–17	NA			NA			426	8.19	(4.37–15.36)
18–24	14	0.37	(0.18–0.80)	197	2.25	(1.28–3.95)	271	2.58	(1.48–4.52)
25–44	28	0.89	(0.41–1.96)	238	1.73	(0.85–3.50)	299	2.09	(1.04–4.22)
45–64	24	2.20	(0.95–5.11)	158	3.21	(1.50–6.84)	192	3.95	(1.86–8.41)
65+	8	2.64	(0.96–7.29)	80	2.28	(1.05–4.94)	90	3.59	(1.66–7.75)
Sex									
Female	27	1.27	(0.70–2.28)	202	1.80	(1.12–2.88)	333	3.03	(1.91–4.81)
Male	49	1.21	(0.78–1.88)	489	3.71	(2.61–5.28)	985	6.43	(4.54–9.10)

COVID: coronavirus disease; VAERS: vaccine adverse event reporting system; mRNA: messenger ribonucleic acid; ROR: reporting odds ratio; NA: not available.

## Data Availability

Publicly available datasets were analyzed in this study. These data can be found here: https://vaers.hhs.gov/ (accessed on 31 August 2021).
